# Effective treatment of refractory monoclonal gammopathy‐associated pure red cell aplasia with isatuximab, pomalidomide and dexamethasone

**DOI:** 10.1002/jha2.964

**Published:** 2024-06-17

**Authors:** Christian Sebastian Michel, Eva Marie Fehr, Hildegard Nolte, Joachim Beck, Andreas Kreft, Katharina Theresa Rauschkolb‐Olk, Bjoern Jacobi, Matthias Theobald, Markus Munder

**Affiliations:** ^1^ Department of Hematology and Medical Oncology University Medical Center of the Johannes Gutenberg University Mainz Germany; ^2^ Department of Pathology University Medical Center of the Johannes Gutenberg University Mainz Germany; ^3^ Institute of Clinical Chemistry and Laboratory Medicine University Medical Center of the Johannes Gutenberg University Mainz Germany

**Keywords:** MGUS, multiple myeloma, pure red cell aplasia

## Abstract

Monoclonal gammopathy‐associated pure red cell aplasia (MG‐PRCA) is characterized by the absence or pronounced hypoplasia of erythroid precursors in the bone marrow, causing reticulocytopenia and a normocytic, normochromic anaemia in a patient with a monoclonal plasma cell dyscrasia. We report here on the successful treatment of MG‐PRCA with isatuximab, pomalidomide, and dexamethasone after multiple lines of immunosuppressive and anti‐plasma cell‐directed treatments.

## 1 CASE REPORT

Monoclonal gammopathy‐associated pure red cell aplasia (MG‐PRCA) is part of the spectrum of monoclonal gammopathies of clinical significance [1]. In this heterogeneous group of diseases, a monoclonal paraprotein, secreted by a B‐cell or plasma cell clone, is thought to be responsible for the impairment of organ function, with monoclonal gammopathy of renal significance as the most widely known subtype [2]. Only few clinical cases have been reported so far for MG‐PRCA and there are no treatment guidelines for patients with this rare disease [3, 4, 5, 6].

The 44‐year‐old male patient presented for the first time in February 2020 in our outpatient department with the diagnosis of a refractory PRCA. Previous treatment lines included bortezomib and dexamethasone (Vd; Apr 2016–Aug 2016 and Dec 2016–Feb 2017) with only a temporary response of reticulocytes, followed by lenalidomide and dexamethasone (Rd) (05/2017‐09/2017), cyclosporine (Nov 2017–Feb 2018), and alemtuzumab (May 2019).

The patient presented with severe fatigue, anaemia and significant transfusion dependence with five packed red blood cells per month. Transfusion dependence had persisted with only short‐term intervals of stabilized haemoglobin over almost 5 years.

**FIGURE 1:**
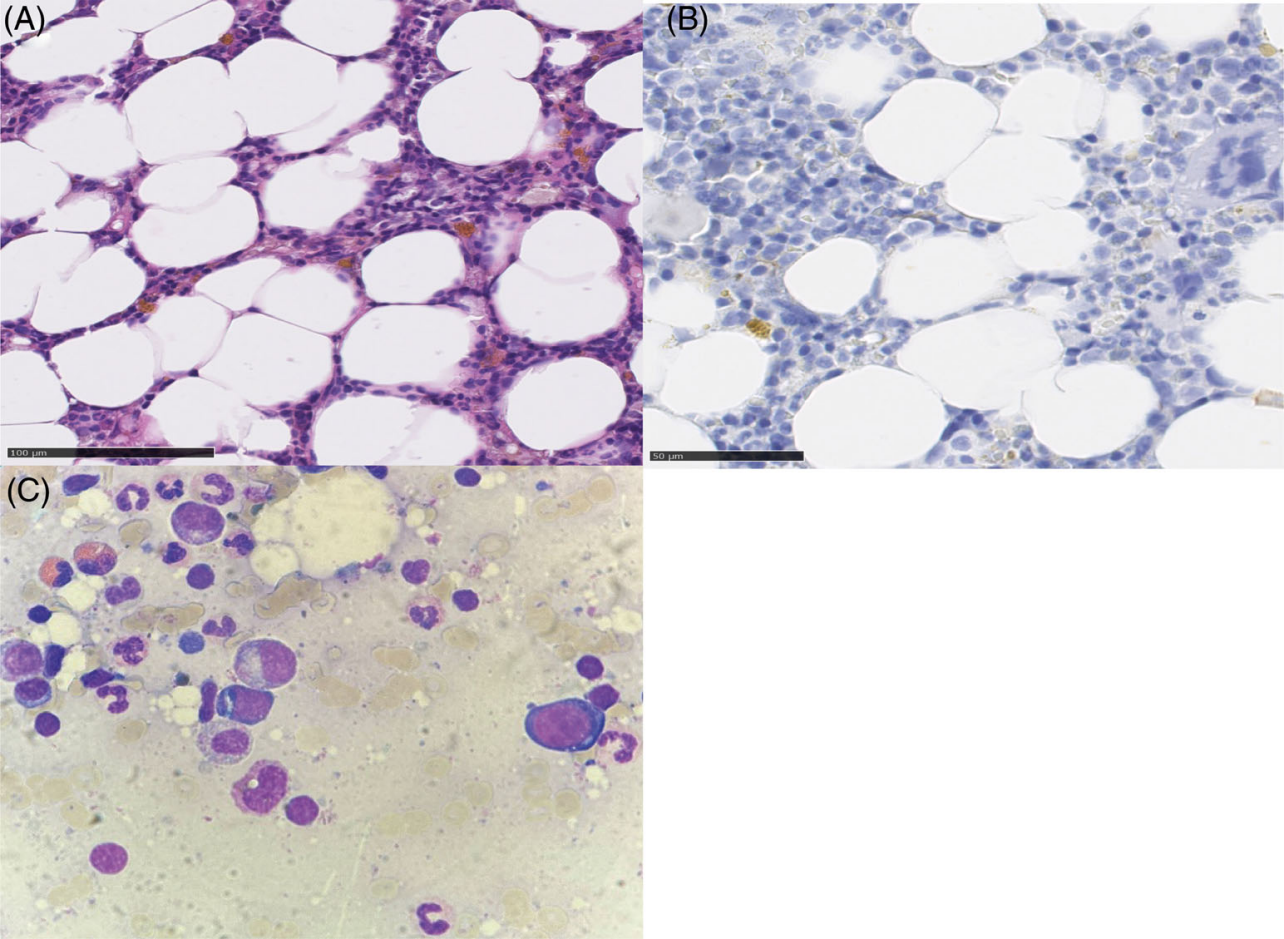
Morphological features of bone marrow biopsy and aspirate pretreatment. (A) Core biopsy with hematoxylin and eosin (H&E) staining (scale bar 100 µm). (B) Core biopsy with E‐Cadherin immunohistochemistry staining (scale bar 50 µm) highlights significantly reduced erythroid differentiation. (C) Bone marrow aspirate (Wright Giemsa, original magnification x100) showing missing differentiation beyond the proerythroblast stage.

**FIGURE 2:**
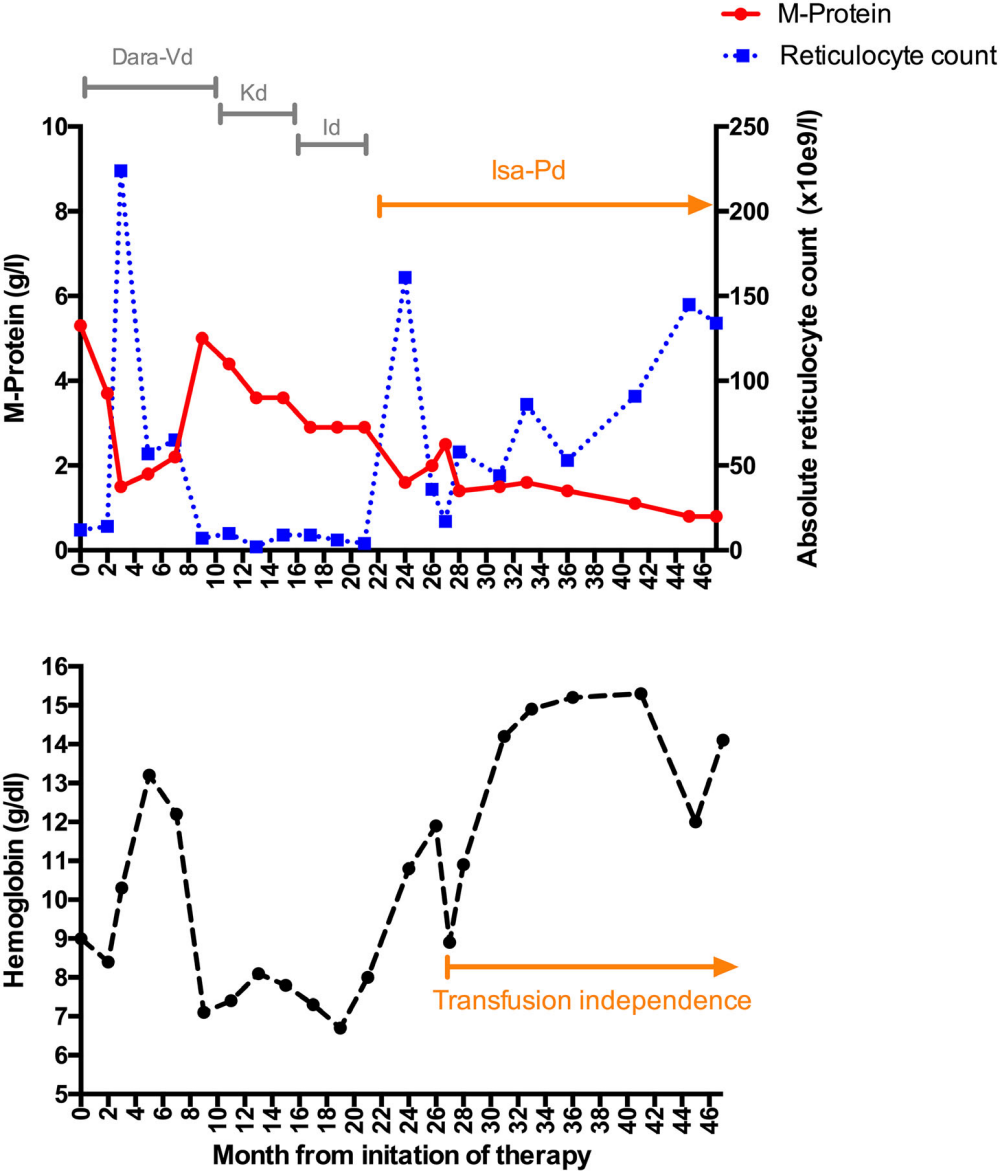
Therapeutic timeline with M‐protein, absolute reticulocyte count and haemoglobin level since treatment started in our department. (A) The sequence of treatments (Dara‐Vd, Kd, ID and Isa‐Pd) in relationship to M‐protein and absolute reticulocyte counts. (B) Time course of hemoglobin level showing transfusion independence after initiation of therapy with isatuximab, pomalidomide, and dexamethasone (Isa‐Pd).

Initial laboratory results showed an absolute reticulocyte count of 12/nL (reticulocyte production index 0.09), normocytic, normochromic anaemia (Hb 9 g/dL) and a monoclonal gammopathy immunoglobulin G type lambda with a serum monoclonal protein (M‐protein) of 6 g/L and serum‐free lambda/kappa ratio of 2.3. Urine protein electrophoresis and immunofixation were positive for lambda light chains, but unquantifiable with regards to light chain excretion. Previously measured erythropoietin level was elevated with 877 mIU/mL (normal range 3.7–29.5mIU/mL). Bone marrow biopsy revealed a monoclonal, lambda‐restricted plasma cell infiltration of less than 5%. Erythropoesis was completely absent beyond the proerythroblast differentiation stage (Figure 1). Cytogenetic analysis demonstrated translocation t(11;14) in CD138‐sorted plasma cells. We hypothesized that a maximally effective treatment targeting the monoclonal plasma cells should reduce or terminate paraprotein‐related phenomena and overcome the refractory PRCA. Based on the previous temporary response towards Vd, we decided to reexpose the patient towards this treatment, augmented with the anti‐CD38 monoclonal antibody daratumumab (Dara‐Vd) [7]. After three cycles of Dara‐Vd, a M‐protein reduction to 1.5 g/L (PR) and a dramatic erythropoetic response with a strong increase in the reticulocyte count (224/nL) were noted (Figure 2). Erythrocyte transfusion independence was achieved. After eight cycles of Dara‐Vd, treatment was continued with daratumumab monotherapy analogous to the CASTOR trial [7]. Unfortunately, under daratumumab monotherapy serum M‐protein increased again to 5 g/L and the reticulocyte count dropped to 7/nL with consecutive necessity for erythrocyte transfusions. Treatment was switched to carfilzomib and dexamethasone (Kd), which led to a moderate decrease of the paraprotein (minimal: 3.6 g/L) but did not affect reticulocyte counts (9/nL) [8]. A switch to the oral proteasome inhibitor ixazomib with dexamethasone (Id) induced a further slight decrease of the serum monoclonal protein, but only a short‐term moderate response in the reticulocyte count (34/nL) without effect on transfusion dependence [9].

After seven lines of immunosuppressive or anti‐plasma cell‐directed treatment, we switched treatment to isatuximab, pomalidomide, and dexamethasone (Isa‐Pd), based on the ICARIA trial protocol [10]. Already after the first cycle of treatment, serum M‐protein dropped from 2.9 to 1.6 g/L and reticulocyte count increased steeply from 4/nL to 161/nL. After 3 months of treatment, the pomalidomide dose had to be reduced from 4 to 2 mg due to severe diarrhea, followed by an increase of M‐protein level to 2.5 g/L and a decrease of the reticulocyte count to 17/nL. By adding loperamide, diarrhea was controlled and pomalidomide dose could be increased stepwise to 4 mg daily with a recurrence of reticulocytosis. The patient is currently still on Isa‐Pd treatment with continuously decreasing M‐protein, normalized hemoglobin and transfusion independence since almost 2 years (Figure 2).

The characteristic feature of MG‐PRCA is the presence of erythroid hypoplasia with erythroid maturation arrest in the bone marrow. This finding is in sharp contrast to the much more common plasma cell disease of multiple myeloma (MM). MM patients usually have the full spectrum of erythroid maturation. The anaemia associated with MM is often a result of a large bone marrow replacement by clonal plasma cells, suppression of erythropoietic differentiation via soluble factors secreted by the plasma cells, iron dysregulation with increased hepcidin levels secondary to interleukin‐6 and BMP‐2 or decreased erythropoietin levels due to myeloma‐induced renal insufficiency [11, 12, 13]. Unfortunately, the underlying pathogenetic mechanism leading to the inhibition of the erythroid lineage in MG‐PRCA is still unclear.

The clinical course of this patient is remarkable for the obvious inverse correlation of the amount of paraprotein and reticulocytosis: lowering of the M‐protein roughly below 2 g/L by efficient targeting of the plasma cell clone is followed by reticulocytosis and erythroid expansion with transfusion independence (Figure 2). We hypothesize that the M‐protein mediated binding of an unknown target antigen on early erythropoetic precursor cells below a certain M‐protein concentration threshold does not lead to effective immune‐mediated destruction or functional impairment of erythroid precursors.

This case report highlights several clinically relevant aspects: (i) The workup of acquired PRCA should always include screening for monoclonal gammopathy, that is, serum and urine immunofixation as well as serum‐free light chain analysis. (ii) Patients with potential MG‐PRCA should be discussed interdisciplinarily to exclude other potential PRCA‐inducing underlying diseases or triggering factors. (iii) A plasma cell‐directed therapeutic approach should be offered to patients, in which PRCA is clinically relevant and its association with the monoclonal gammopathy is deemed likely. (iv) Therapy should aim for efficient reduction of the M‐protein since the termination of the erythropoiesis‐inhibitory activity might be seen only after a certain M‐protein threshold is reached. In summary, therapeutic nihilism in patients with MG‐PRCA should definitively be avoided.

## AUTHOR CONTRIBUTIONS

Christian Sebastian Michel, Bjoern Jacobi, Matthias Theobald and Markus Munder wrote the manuscript. Christian Sebastian Michel, Eva Marie Fehr, Hildegard Nolte, Joachim Beck and Markus Munder treated the patient. Katharina Theresa Rauschkolb‐Olk provided laboratory data, and Andreas Kreft and Christian Sebastian Michel provided imaging data. All authors critically reviewed and approved the manuscript.

## CONFLICT OF INTEREST STATEMENT

CSM reports consulting or advisory roles for Janssen, Bristol‐Myers Squibb, Oncopeptipdes and GSK. MM reports consulting or advisory role for Janssen, Takeda, Amgen, Bristol‐Myers Squibb, Sanofi, GSK, Stemline and Oncopeptides; honoraria from Janssen, Takeda, Amgen, Bristol‐Myers Squibb, Sanofi, GSK and AbbVie. The remaining authors declare no conflict of interest.

## FUNDING INFORMATION

No sources of funding are applicable to this article.

## ETHICS STATEMENT

Data were collected in accordance with institutional protocols.

## PATIENT CONSENT STATEMENT

Patient consent was obtained and available on request.

## CLINICAL TRIAL REGISTRATION

The authors have confirmed clinical trial registration is not needed for this submission.

## Data Availability

The data that support the findings of this study are available on request from the corresponding author CSM. The data are not publicly available due to restrictions, for example, they contain information that could compromise the privacy of research participants. The participant of this study gave written consent for his data to be shared anonymously in public.
